# A Prospective Multi-Center Observational Study of Children Hospitalized with Diarrhea in Ho Chi Minh City, Vietnam

**DOI:** 10.4269/ajtmh.14-0655

**Published:** 2015-05-06

**Authors:** Corinne N. Thompson, My V. T. Phan, Nguyen Van Minh Hoang, Pham Van Minh, Nguyen Thanh Vinh, Cao Thu Thuy, Tran Thi Thu Nga, Maia A. Rabaa, Pham Thanh Duy, Tran Thi Ngoc Dung, Voong Vinh Phat, Tran Vu Thieu Nga, Le Thi Phuong Tu, Ha Thanh Tuyen, Keisuke Yoshihara, Claire Jenkins, Vu Thuy Duong, Hoang Le Phuc, Pham Thi Ngoc Tuyet, Nguyen Minh Ngoc, Ha Vinh, Nguyen Tran Chinh, Tang Chi Thuong, Ha Manh Tuan, Tran Tinh Hien, James I. Campbell, Nguyen Van Vinh Chau, Guy Thwaites, Stephen Baker

**Affiliations:** The Hospital for Tropical Diseases, Wellcome Trust Major Overseas Programme, Oxford University Clinical Research Unit, Ho Chi Minh City, Vietnam; Centre for Tropical Medicine, Nuffield Department of Clinical Medicine, Oxford University, United Kingdom; The London School of Hygiene and Tropical Medicine, London, United Kingdom; The Wellcome Trust Sanger Institute, Hinxton, Cambridge, United Kingdom; Centre for Immunity, Infection and Evolution, University of Edinburgh, Edinburgh, United Kingdom; The Institute of Tropical Medicine, Nagasaki University, Nagasaki, Japan; Gastrointestinal Bacteria Reference Unit, Public Health England, London, United Kingdom; Children's Hospital 1, Ho Chi Minh City, Vietnam; Children's Hospital 2, Ho Chi Minh City, Vietnam; Hospital for Tropical Diseases, Ho Chi Minh City, Vietnam

## Abstract

We performed a prospective multicenter study to address the lack of data on the etiology, clinical and demographic features of hospitalized pediatric diarrhea in Ho Chi Minh City (HCMC), Vietnam. Over 2,000 (1,419 symptomatic and 609 non-diarrheal control) children were enrolled in three hospitals over a 1-year period in 2009–2010. Aiming to detect a panel of pathogens, we identified a known diarrheal pathogen in stool samples from 1,067/1,419 (75.2%) children with diarrhea and from 81/609 (13.3%) children without diarrhea. Rotavirus predominated in the symptomatic children (664/1,419; 46.8%), followed by norovirus (293/1,419; 20.6%). The bacterial pathogens *Salmonella*, *Campylobacter*, and *Shigella* were cumulatively isolated from 204/1,419 (14.4%) diarrheal children and exhibited extensive antimicrobial resistance, most notably to fluoroquinolones and third-generation cephalosporins. We suggest renewed efforts in generation and implementation of policies to control the sale and prescription of antimicrobials to curb bacterial resistance and advise consideration of a subsidized rotavirus vaccination policy to limit the morbidity due to diarrheal disease in Vietnam.

## Introduction

Childhood diarrhea remains a serious global public health issue, with an estimated 1.7 billion infections and 0.7 million deaths in children under 5 years annually, most of which occur in industrializing regions.^1^,^2^ Rotavirus (RoV) and norovirus (NoV) are together responsible for an estimated 40% of severe diarrhea in children in low- and middle-income countries,^3^ with bacterial pathogens *Shigella* spp., *Campylobacter* spp., and *Salmonella* spp. commonly identified as well.^4^ *Cryptosporidium*, a protozoan, has also recently been found to cause a significant proportion of moderate to severe diarrhea in children < 5 years of age in resource-poor countries.^4^ Diagnosis and treatment of diarrheal disease in such settings, however, is hampered by a lack of laboratory capacity, lack of specific clinical indicators, overuse of antimicrobial therapy, and a resultant increase in antimicrobial resistance.^5^

Diarrhea is a significant cause of morbidity in children in Ho Chi Minh City (HCMC), Vietnam,^6^,^7^ yet there is limited data on etiology, clinical features, and prevalence of antimicrobial resistance among children hospitalized with diarrhea in this setting. HCMC is a densely populated, rapidly industrializing city that is home to over 8 million people in southern Vietnam.^8^ Rampant antimicrobial usage in the community has led to alarming reports of third-generation cephalosporin and fluoroquinolone resistance among pathogenic and commensal gastrointestinal bacteria.^9^,^10^ Furthermore, appropriate treatment and prevention strategies may be hindered by a lack of distinguishing, pathogen-specific clinical characteristics. To address gaps in knowledge regarding hospitalized pediatric diarrhea in HCMC, we conducted a cross-sectional hospital-based study aiming to describe etiological and clinical features, epidemiological characteristics, and antimicrobial susceptibility profiles of pediatric diarrheal disease in this rapidly developing southeast Asian city.

## Materials and Methods

### Study sites and ethical approval.

This prospective, hospital-based study was conducted at three hospitals in HCMC: Children's Hospital 1 (CH1), Children's Hospital 2 (CH2), and the Hospital for Tropical Diseases (HTD). CH1 and CH2 are the largest pediatric hospitals (1,500 beds each) in HCMC while HTD is a 500-bed referral hospital in southern Vietnam. The Scientific and Ethics Committee of CH1, CH2, HTD and the University of Oxford Tropical Research Ethics Committee (OxTREC No. 0109) approved this study. Written informed consent was required from parents or legal guardians prior to participation in the study.

### Enrollment procedures and inclusion/exclusion criteria.

Children ≤ 60 months of age who were admitted with acute diarrheal disease to the gastrointestinal wards of the three hospitals from May 2009 to April 2010 were considered for inclusion in this study as diarrheal cases. Inclusion criteria were diarrhea as the primary reason for admission (defined as three or more loose stools or at least one bloody loose stool within a 24-hour period, according to the World Health Organization [WHO] guidelines),^11^ resident within the districts of HCMC, and no antimicrobial treatment within 3 days prior to study enrollment. We excluded children with a history of antimicrobial treatment in an effort to conserve resources as we rarely isolate any organisms from such patients in this setting (James Campbell, unpublished data). There were no additional exclusion criteria. The first five patients that met the inclusion criteria were enrolled at each study site on weekdays because of resource and staff limitations.

Children of the same age range (0–60 months) attending CH1 or CH2 for a health check or for other gastrointestinal issues unrelated to diarrhea, gastroenteritis, or other infectious diseases between March and December 2010 were invited for enrollment as hospital-based, non-diarrheal controls. Inclusion criteria included living within the districts of HCMC, no antimicrobial use within 3 days prior to hospital admission, and no history of diarrhea or respiratory illness within 7 days of study enrollment. There were no additional exclusion criteria.

### Sample collection and microbiological methods.

A stool specimen was collected in a sterile container from each enrollee as soon as possible and prior to any prescribed antimicrobial treatment. A specimen was collected within 24 hours of hospital admission to limit detection of nosocomial infection. Classical microbiological culturing was performed on all collected fresh stool samples on the day of sampling to isolate common diarrheal bacteria including *Shigella* spp., *Salmonella* spp., *Campylobacter* spp., *Yersinia* spp., *Plesiomonas* spp., and *Aeromonas* spp. as described previously.^12^ Specific serotypes of *Shigella* spp. and *Salmonella* spp. were identified by slide agglutination with antigen grouping sera and monovalent antisera, and *Campylobacter jejuni* was differentiated from *Campylobacter coli* by the hippurate hydrolysis test as previously described.^12^ A fresh smear of fecal specimen was prepared in phosphate buffered saline to examine the presence of *Giardia lamblia*, *Entamoeba histolytica*, and *Cryptosporidium* cysts.^12^

### Antimicrobial susceptibility and extended-spectrum β-lactamase testing.

The minimum inhibitory concentrations (MICs) of bacterial isolates were determined by E-test (AB Biodisk, Solna, Sweden) using the disc diffusion method following the Clinical and Laboratory Standards Institute (CLSI) guidelines.^13^ Twelve antimicrobials were tested: ciprofloxacin (CIP), ceftriaxone (CRO), ceftazidime (CAZ), amoxicillin-clavulanic acid (AUG), erythromycin (ERY), ofloxacin (OFX), ampicillin (AMP), trimethoprim-sulfamethoxazole (SXT), azithromycin (AZT), chloramphenicol (CHL), gatifloxacin (GA), and nalidixic acid (NA). The production of extended-spectrum β-lactamases (ESBL) was detected using the double-disc synergy test: isolates with an increase in diameter of inhibitory zone of ≥ 5 mm because of the synergy of clavulanate were considered to be ESBL positive.^14^

### Molecular detection of RoV and NoV.

For molecular testing, total viral RNA was extracted, reverse transcribed into cDNA as previously described,^15^ and used to detect RoV and NoV by reverse transcriptase polymerase chain reaction (RT-PCR). RoV detection was performed targeting the outer capsid genes (VP7 and VP4),^16^ while NoV genogroup I (GI) and II (GII) were identified in separate PCR reactions targeting the conserved region overlapping open reading frame (ORF) 1–2.^17^ All PCR amplicons were visualized on 2% agarose gels under ultraviolet (UV) light after staining with 3% ethidium bromide.

### Molecular detection of pathogenic *Escherichia coli*.

A total of 360 stool samples (210 from symptomatic cases and 150 from non-diarrheal controls) that were parasite negative and culture negative for all tested bacteria and RT-PCR negative for RoV and NoV were randomly selected to screen for the presence of pathogenic *E. coli* variants. Total nucleic acid was extracted from these samples using an automated MagNA Pure 96 nucleic extraction system (Roche Applied Sciences, West Sussex, United Kingdom) according to the manufacturer's recommendations. Multiple PCR reactions were performed directly on the extracted DNA to detect enterohemorrhagic *E. coli* (EHEC), enteropathogenic *E. coli* (EPEC), enteroinvasive *E. coli* (EIEC), enterotoxigenic *E. coli* (ETEC), and enteroaggregative *E. coli* (EAEC). Multiplex real-time PCRs were performed to detect EHEC and EPEC in one duplex reaction^18^ and to detect EIEC and EAEC in an additional duplex. ETEC was detected and classified as ETEC-LT (heat labile), ETEC-ST (heat stable), or ETEC-LT/ST using independent conventional PCR under previously described conditions.^19^,^20^ PCR amplicons were visualized on 2% agarose gels under UV light after staining with 3% ethidium bromide.

### Clinical and demographic data.

A simple case report form (CRF) was completed for each enrolled patient by study clinicians to obtain data regarding symptoms, disease duration, and treatment regimens as per the standard care at the study hospitals. Study staff administered a confidential questionnaire detailing demographic, socioeconomic, and behavioral characteristics to all enrolled individuals. Average rainfall and temperature data for HCMC were obtained from the Vietnam Southern Regional Hydro-Meteorological Station. The nutritional status of all enrolled children was expressed as the weight-for-age Z (WAZ) score based on WHO growth standards^21^; children with a WAZ score value below −2 were considered malnourished.^22^

### Statistical analyses.

Tabulations of demographic, clinical, and laboratory characteristics among cases and non-diarrheal controls were performed with STATA 9.2 (StataCorp, College Station, TX) and compared using the χ^2^ test, Fisher's exact test, or Mann–Whitney *U* test as appropriate. Two-sided *P* values ≤ 0.05 were considered statistically significant.

## Results

### Demographic characteristics.

Over the study period, 1,419 diarrheal cases (referred hereafter as cases) and 609 non-diarrheal control children (referred hereafter as non-diarrheal controls) were enrolled; the demographic characteristics of the cases and non-diarrheal controls are shown in Table 1. Both the cases and non-diarrheal controls were more frequently male (64% and 53%, respectively) and had a combined median age of 12 months (interquartile range [IQR]: 8–20 months). The non-diarrheal controls were more likely to have a poor WAZ score than cases (13% versus 7%; *P* < 0.001, χ^2^ test). Of cases and the non-diarrheal controls, 70% were frequently breast-fed as infants, yet the regular use of milk formula and probiotics was more common among the non-diarrheal controls (82%; 498/609 and 64%; 304/473, respectively) than the cases (58%; 819/1,419 and 14%; 121/861, respectively) (*P* < 0.001 for each comparison, χ^2^ test). The majority of families of the enrollees (> 80%) resided within the urban districts of HCMC, as opposed to the peri-urban/rural areas. In addition, cases were more likely to report a lower income than controls. The households of more than half of all enrolees used a government pipeline as their major household water source; there were no significant differences in water source between cases and the diarrheal controls.

### The prevalence of enteric pathogens.

At least one known enteric pathogen was identified in 75.2% (1,067/1,419) of stool samples from the cases and in 13.3% (81/609) of stool samples from the non-diarrheal controls (Table 2). The majority of enrollees with an isolate-positive stool sample (cases 91%; 970/1,067 and non-diarrheal controls 94%; 76/81) were infected with a single pathogen from the screened panel (see Methods). Of cases, 97 (9%) were infected with two pathogens; including combinations of bacteria (0.4%; *N* = 5), viruses (2.3%; *N* = 32), or virus and bacteria (4.2%; *N* = 60). RoV and NoV were identified in 46.8% (664/1,419) and 20.6% (293/1,419) of all cases, respectively (Table 2). The bacterial genera *Salmonella*, *Shigella*, and *Campylobacter* were isolated from 57 (4.0%), 48 (3.4%), and 31 (2.2%) cases, respectively. Of patients with an isolated bacterial pathogen, 67 (33%) cases had an additional pathogen (Table 2). No *Cryptosporidium* isolates were identified.

In contrast to the dominance of viral infections among cases, bacterial pathogens were identified in a greater proportion than viruses among the non-diarrheal controls. *Salmonella* was the most commonly identified bacterial pathogen of the 609 non-diarrheal controls, isolated from a total of 39 (6.4%) stool samples (Table 2), while *Campylobacter* was identified in 16 (2.6%). None of the non-diarrheal controls were culture positive for *Shigella* spp. In addition, NoV and RoV were isolated from 17/609 (2.8%) and 13/609 (2.1%), respectively, non-diarrheal controls.

Pathogenic *E. coli* were detected by PCR amplification in 34% (72/210) and 55% (82/150) subset of randomly selected stool samples from the cases and non-diarrheal controls, respectively, in which no other pathogen was identified. As shown in Table 3, EPEC was the most common pathogenic *E. coli* variant detected, found more frequently in non-diarrheal controls (51%; 76/150) than in diarrheal children (19%; 39/210). However, there was significantly more atypical EPEC detected in non-diarrheal controls than in diarrheal cases (50% versus 18%; *P* < 0.001, χ^2^ test). EAEC was the second most common *E. coli* variant detected, identified in the stools of a similar proportion of cases (9%; 18/210) and non-diarrheal controls (11%; 17/150). EIEC and ETEC were identified less frequently, but both were more commonly isolated in children with diarrhea as shown in Table 3.

### Clinical manifestations.

The clinical characteristics, type of diarrheal stool, and the presence of red and white blood cells in stool (by microscopy) were recorded for all diarrheal cases on admission (Table 4). Loose watery diarrhea was the most commonly recorded stool type (79%; 1,118/1,419), which was most prevalent among children who had a viral enteric pathogen in their stool (89%; 766/863). Approximately half of the cases infected with *Shigella* (49%; 23/47), *Campylobacter* (58%; 18/31), and *Salmonella* (49%; 28/57) presented with visible blood or mucus in their stool. The majority of all cases had moderate (37.2–39°C) (52%; 744/1,419) or high fever (> 39°C) (22%; 312/1,419) in addition to vomiting (78%; 1,100/1,419).

Symptomatic children with a viral pathogen in their stool were more likely to present with dehydration and vomiting and to have had diarrhea for a longer period prior to hospitalization, than those with a bacterial pathogen, as shown in Table 4. The symptomatic cases with a bacterial pathogen in their stool were more likely to present with abdominal pain, severe fever and to have blood cell-positive stool smears. The median age of cases was comparable when stratified by the pathogen(s) found in the stools, with the exception of children with a *Shigella* infection (median: 31 months; IQR: 20–36 months), who were significantly older than cases with other enteric pathogens identified in their stool (median: 13 months; IQR: 8–19 months) (*P* < 0.001, Mann–Whitney *U* test).

### Diarrheal treatment regimes.

Cases with a confirmed bacterial infection were more likely to be prescribed an antimicrobial on presentation (74%; 106/143) than those with a viral infection (38%; 324/862) (*P* < 0.001, χ^2^ test), where the prescription of antimicrobials was determined by clinical presentation before etiological investigation. Approximately half (48%; 29/60) of those with a combined bacterial/viral infection were prescribed an antimicrobial, and 60% (210/352) of those with diarrhea of unknown origin were treated with antimicrobials. Children with visible blood and/or mucus in their stool were prescribed antimicrobials frequently (75%; 226/301) as were children with high fever (68%; 214/313). The most commonly prescribed groups of antimicrobials were fluoroquinolones (61%; 438/714) and third-generation cephalosporins (22%; 160/714). Zinc and probiotics were prescribed slightly more frequently to those who had a confirmed viral infection (74%; 635/862 and 69%; 591/862, respectively) than those who had a bacterial infection (66%; 95/143 and 62%; 88/143, respectively).

### Spatial and temporal distribution of enteric pathogens.

**Figure 1.**
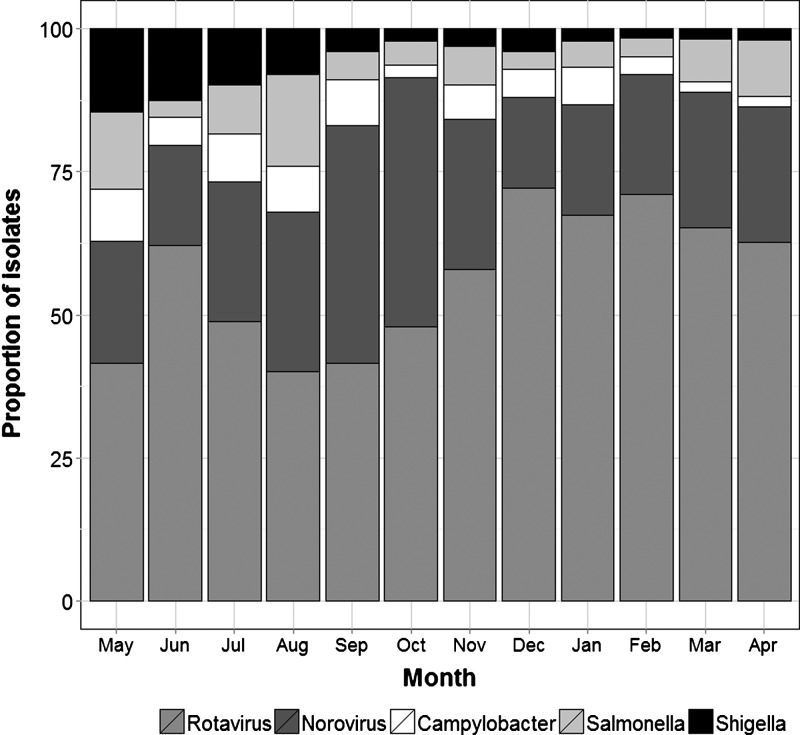
Proportion of various diarrheal etiologies among total isolates identified per month over the course of the study period. The proportion of isolates identified as rotavirus (medium gray), norovirus (dark gray), *Campylobacter* spp. (white), *Salmonella* spp. (light gray), and *Shigella* spp. (black) identified over each month of the study period (May 2009–April 2010) are shown as a stacked bar chart.

Using the available location data from the diarrheal patients, we found no association between the relative proportion of each pathogen and population density or urban/peri-urban locations, with RoV and NoV predominating in all districts of the city. In addition, we found no association between the frequency of cases caused by any of the identified pathogens and monthly mean temperature or rainfall, with the exception of a positive correlation between rainfall and the isolation of *Shigella* spp. (Spearman's correlation coefficient [*r*] = 0.592, *P* = 0.043). RoV was identified more frequently in the drier months (January–March), although the relationship was not significant (*r* = −0.417, *P* = 0.178) (Figure 1).

### Antimicrobial susceptibility.

The MIC distribution of the *Campylobacter*, *Shigella*, and *Salmonella* isolated from the cases against selected antimicrobials are shown in Table 5. The majority (≥ 80%) of the *Campylobacter* isolates exhibited resistance to NA and CIP, with only 8% (5/64) resistant to ERY. A large proportion (≥ 75%) of the 62 *Shigella* isolates were also resistant to NA and CRO. However, the *Salmonella* isolates were comparatively more susceptible to both fluoroquinolones and third-generation cephalosporins, with few (≤ 20%) isolates displaying resistance to CRO, CIP, or NA. Notably, the *Campylobacter* and *Shigella* isolates were relatively susceptible to CHL, with resistance identified in ≤ 2% of *Campylobacter* isolates, and in ≤ 7% of *Shigella* isolates. Many (40%) of the *Salmonella* isolates exhibited resistance to CHL. The majority (≥ 90%) of the *Campylobacter* and *Shigella* isolates demonstrated resistance to ≥ 3 classes of antimicrobials, and a high proportion of the *Shigella* isolates were ESBL positive (75%; 47/62). In contrast, a negligible proportion of *Salmonella* isolates exhibited ESBL activity (3%; 2/78).

In general, a lower proportion of pathogens isolated from stools of the non-diarrheal controls demonstrated antimicrobial resistance compared with isolates from symptomatic cases. Specifically, all *Salmonella* isolates from the non-diarrheal controls were susceptible to CIP, CRO, and CAZ. Resistance to CHL was low (2.6%; 1/39). However, many of the *Campylobacter* isolates from the non-diarrheal control stool samples were resistant to CIP (68.7%; 11/16), NA (62.5%; 10/16), and CHL (18.7%; 3/16).

## Discussion

This is one of the few studies addressing the causative agents of diarrheal disease in southern Vietnam. Using the described methods, we were able to detect at least one known diarrheal pathogen in 75% of all diarrheal cases, which is comparable to the findings by previous case–control studies conducted in the north of Vietnam (67%; *N* = 587),^23^ and in seven developing countries in Africa and Asia within The Global Enteric Multicenter Study (GEMS) (83%; *N* = 9,439).^4^ In this study, the remaining 25% cases in which we could not isolate a pathogen may correspond with disease caused by alternative nontargeted pathogens, limited diagnostic sensitivity, unreported pretreatment with antimicrobials, or other causes, such as food allergy, malabsorption, or maldigestion.^24^

Diarrheal disease in young children in industrialized countries is generally considered to be caused by viral pathogens, while bacterial and parasitic diarrheal agents are generally considered more prevalent in industrializing countries.^25^ Here, we identified viral pathogens more frequently than bacterial pathogens in diarrheal cases, potentially reflecting the effect of the recent economic transition in Vietnam on the epidemiology of enteric pathogens in HCMC.^7^ Furthermore, the large proportion of children hospitalized with RoV-induced diarrhea in this setting predicts that uptake of RoV vaccine would have a considerable impact on the diarrheal disease burden in this population. In Vietnam, the current cost of RoV immunization is prohibitive (approximately $75 for a full course of RotaTeq [Merck, West Point, PA] or RotaRix [GSK, Middlesex, UK]) and neither of these licensed vaccines are currently included in Vietnam's Extended Program of Immunization (EPI) schedule. We therefore suggest that the integration of RoV vaccine into the EPI schedule should be considered as a matter of necessity.^26^ An alternate way forward for RoV vaccination in Vietnam would be through regional mass production of a generic vaccine, such as the live-attenuated monovalent G1P[8] (Rotavin-M1, National Institute of Hygiene and Epidemiology, Hanoi, Vietnam) vaccine that has recently undergone trials in Vietnam.^27^,^28^ This approach may reduce the cost of RoV immunization to the Vietnamese health service, permitting a greater national coverage and thus greater impact.

The variable pathogenicity of *E. coli* pathovars in addition to the cost and technical difficulties in diagnostic detection of this group have greatly hindered the understanding of disease epidemiology and pathogenesis of this bacterial species in childhood diarrhea, particularly in resource-limited settings. We were able to detect the presence of five different *E. coli* pathogenic variants from a random cross section of stools from cases and non-diarrheal controls without an alternate identified etiology by PCR amplification. We found that atypical EPEC was the most common variant in both children with and without diarrhea. Recent evidence from the GEMS study suggested that atypical EPEC was not associated with moderate-to-severe diarrhea,^4^ confirmed by our findings as non-diarrheal controls were more likely to be infected with this variant. This association with EPEC has been previously observed.^29^ EPEC has also been reported to be associated with a more persistent clinical diarrheal syndrome rather than the acute diarrheal syndrome that was assessed here.^30^ We additionally found that ETEC-LT was predominant and significantly associated with cases in comparison to non-diarrheal controls in this study. However, findings from GEMS suggest that ETEC-ST is a significant diarrheal pathogen and ETEC-LT is not a significant cause of moderate-to-severe diarrhea.^4^ We conclude that this discrepancy reflects the differing epidemiology of ETEC between settings, which may be related to different routes of transmission, behavioral risk factors, and water quality.^31^

The rate of pathogen detection in our non-diarrheal control population was much lower than that identified in the GEMS study (13% versus 72%).^4^ The GEMS study screened for a wider variety of pathogens, often using more sensitive assays, which could in part explain the difference. The exclusion of children who had received recent antimicrobial treatment may have biased our non-diarrheal control population as well. Also, the controls were more likely to report higher socioeconomic status indicators, and are thus likely from a different epidemiological population than the cases, which also may explain the low rate of pathogen detection in this group. However, one of the more notable findings from this study was that *Salmonella* spp. was detected in a similar proportion in cases and non-diarrheal controls (6%). *Campylobacter* spp., although more common in cases (5%), was also identified in 3% non-diarrheal controls. Recent case–control studies conducted in southeast Asia have found a high prevalence of *Campylobacter* spp. and *Salmonella* spp. from healthy non-diarrheal controls in rural Thailand and Cambodia.^32^,^33^ These data imply that asymptomatic/transient infection with these organisms may be substantial in these areas; further research is warranted to determine the clinical relevance and role in disease transmission in the community.

We found remarkable levels of antimicrobial resistance in the pathogens isolated as part of this study; > 90% of *Campylobacter* spp. and *Shigella* and > 50% of *Salmonella* spp. isolates exhibited resistance to three or more classes of antimicrobials. Reduced susceptibility and resistance to broad-spectrum antimicrobials in enteric pathogens is becoming increasingly reported across Asia, and our data support the notion that resistance to multiple antimicrobial groups is common across multiple genera of enteric pathogens.^34^ For example, we found an exceptionally high prevalence of ciprofloxacin-resistant *Campylobacter*, which has been recorded in several Asian countries, including Cambodia, India, and China.^33^,^35^,^36^ Antimicrobial therapy is not generally recommended to treat non-dysenteric diarrhea, although ciprofloxacin is commonly used as a first-line antimicrobial in the case of profuse or bloody mucoid diarrhea.^37^ As routine identification of any causative agents of diarrhea is not performed in hospitals in Vietnam, patients are treated following standard Vietnamese treatment guidelines (ciprofloxacin or ceftriaxone as first- and second-line therapies) based on clinical observations, which may be insensitive in distinguishing viral and bacterial infections. Developing affordable rapid point-of-care diagnosis should be considered to help clinicians choose more appropriate antimicrobial regimens for diarrheal patients in addition to encouraging continued use of rehydration and zinc supplementation.

Ceftriaxone, a third-generation cephalosporin, is recommended by the WHO as an alternative treatment of severe infectious diarrhea and shigellosis.^11^,^37^ Yet the prevalence of ceftriaxone resistance has increased markedly, particularly in *Shigella* spp., in this location in the past decade.^7^,^10^ The first ESBL-mediated ceftriaxone-resistant *Shigella* in southern Vietnam was isolated from a pediatric diarrheal patient in 2001 at the HTD, HCMC.^10^ The prevalence of ceftriaxone-resistant *Shigella* strains found in this study (75%) was triple that of the 2007–2008 period in pediatric diarrheal patients in the same hospital (23%; *N* = 103).^7^ The true prevalence of these resistant *Shigella* strains circulating in the community may be vastly underestimated because of a lack of routine diagnosis and antimicrobial resistance surveillance in the region. Moreover, we postulate that antimicrobial-resistant bacteria are circulating in commensal enteric microbiota in the community at high levels.^9^ This hypothesis, in addition to the high reported rates of antimicrobial usage and resistance in poultry in Vietnam,^38^ would increase the likelihood of antimicrobial resistance gene transfer, thus increasing the rate of emergence of multidrug-resistant strains and limiting effective therapeutic regimes for treating patients with severe or life-threatening bacterial infections.^10^ Developing solutions against antimicrobial resistance is becoming a serious global challenge as we enter the “postantibiotic era.”^39^,^40^ In this context, we reiterate that regulating antimicrobial usage in the community and agriculture as well as improved control of hospital prescription practices may be a critically necessary strategy of slowing the rate of increasing antimicrobial resistance in this location.

Our study has some limitations. First, our non-diarrheal control population may not adequately represent all children without diarrhea in this community. The majority of the non-diarrheal controls were children attending a routine clinic for health checks, which may result in the introduction of biases. Second, we included only the first five patients seen each day; hence the description of seasonal patterns is limited by the recruitment pattern. Next, the proportion of pathogen-positive stool samples may be underestimated because our diagnostic methods targeted a limited group of pathogens, potentially missing other enteric pathogens such as adenovirus, astrovirus, and helminths. In addition, viral etiologies were likely identified more often due to sensitivity differences between PCR-based diagnostics compared with culture techniques used for bacterial pathogens and due to the exclusion of patients who had received recent antimicrobial therapy. The lack of *Cryptosporidium* isolates specifically, given the high prevalence identified in the GEMS study,^4^ is likely due to the poor sensitivity of microscopy.^41^ Finally, our study was hospital based, thus our passive enrollment and case detection was entirely dependent on health-care-seeking behavior. Therefore, our study will inevitably skew results toward the moderate-to-severe end of the disease spectrum, as the bulk of mild infections remain undetected in the community. Notwithstanding these limitations, we suggest that our clinical observations, etiology, and prevalence data are informative and likely to be broadly representative of hospitalized diarrhea in this setting and other economically transitioning regions in southeast Asia.

In conclusion, we identified a known enteric pathogen in 75% cases of hospitalized pediatric diarrhea in HCMC, with RoV and NoV being the most frequently identified. While bacterial pathogens were identified in only 14% cases, alarming rates of antimicrobial resistance to recommended first- and second-line therapies are likely to result in a growing burden of hospitalized diarrhea in young children in this setting. We suggest renewed efforts in generation and implementation of policies to control the sale and prescription of antimicrobials to curb bacterial resistance and advise consideration of a subsidized RoV vaccination policy to limit the morbidity due to diarrheal disease in Vietnam.

## Figures and Tables

**Table 1:** The demographic characteristics of diarrheal and non-diarrheal children

Characteristic	Cases *n* (%)	Non-diarrheal controls *n* (%)	*P* value§
	*N* = 1,419	*N* = 609	
Male sex	905 (63.8)	322 (52.9)	< **0.001**
Median age (IQR) months	13 (8–19)	12 (8–20)	0.711
Poor WAZ*	93 (6.6)	76 (12.5)	< **0.001**
Breast-fed	1,017 (71.7)	465 (76.4)	**0.029**
Day care/nursery school attendance	223 (15.9)	93 (15.4)	0.837
Median household size (IQR)	6.5 (2–31)	6.4 (3–26)	0.445
Income bracket (monthly)			
< $145	422 (29.7)	136 (22.3)	< **0.001**
$145–242	532 (37.5)	211 (34.6)	
$243–483	326 (23)	168 (27.6)	
$484–725	90 (6.3)	61 (10.1)	
> $725	49 (3.5)	33 (5.4)	
Household water source			
Government pipeline	835 (59.0)	359 (59.0)	0.471
Well	501 (35.4)	223 (36.6)	
Other†	81 (5.7)	27 (4.4)	
Residential location‡			
Rural/peri-urban	261 (18.4)	76 (12.5)	**0.001**
Urban	1,158 (81.6)	533 (87.5)	

IQR = interquartile range; WAZ = weight-for-age Z score.

* Weight-for-age Z-score < −2.^21^,^22^

† Other household water sources include rainwater, well water, and water bought from governmental truck dispenser.

‡ Rural/peri-urban and urban districts.

§ *P* values through χ^2^ or Mann–Whitney *U* test as appropriate.

**Table 2:** Enteric pathogens identified in the stools of diarrheal cases and non-diarrheal controls

Organism	Cases *n* (%)	Non-diarrheal controls *n* (%)	*P* value*
	*N* = 1,419	*N* = 609	
Norovirus	241 (17.0)	15 (2.5)	< **0.001**
Rotavirus	590 (41.6)	10 (1.6)	< **0.001**
*Campylobacter*	31 (2.2)	16 (2.6)	0.544
*jejuni*	19 (1.3)	11 (1.8)	0.424
*coli*	12 (0.8)	5 (0.8)	0.955
*Salmonella*	57 (4.0)	34 (5.6)	0.118
Group B	35 (2.5)	12 (2.0)	0.496
Group C	8 (0.6)	0 (0.0)	0.115
Group D	4 (0.3)	1 (0.2)	1.000
spp.	9 (0.6)	21 (3.4)	< **0.001**
*arizonae*	1 (0.1)	0 (0)	1.000
*Shigella*	48 (3.4)	0 (0)	< **0.001**
*flexneri*	4 (0.3)	0 (0)	0.323
*sonnei*	44 (3.1)	0 (0)	< **0.001**
Other bacteria	2 (0.1)	1 (0.2)	1.000
Parasites	1 (0.1)	0 (0)	1.000
Mixed viral RoV/NoV	32 (2.3)	1 (0.2)	< **0.001**
Mixed viral bacterial	60 (4.2)	3 (0.5)	< **0.001**
Mixed bacteria	5 (0.4)	1 (0.2)	0.675
Total	1,067 (75.2)	81 (13.3)	< **0.001**

* *P* value from χ^2^ test or Fisher's exact test, as appropriate; boldface indicates statistical significance.

**Table 3:** The prevalence of pathogenic *Escherichia coli* variants in the stools of diarrheal cases and non-diarrheal controls

*E. coli* variant	Cases *n* (%)	Non-diarrheal controls *n* (%)	*P* value*
	*N* = 210	*N* = 150	
EPEC	39 (18.6)	76 (50.7)	< **0.001**
Typical	1 (0.5)	1 (0.7)	1.000
Atypical	38 (18.1)	75 (50.0)	< **0.001**
EHEC	2 (1.0)	1 (0.7)	1.000
EAEC	18 (8.6)	17 (11.3)	0.471
EIEC	21 (10.0)	3 (2.0)	**0.002**
ETEC	12 (5.7)	2 (1.3)	**0.050**
LT	10 (4.8)	1 (0.7)	**0.029**
ST	1 (0.5)	1 (0.6)	1.000
LT and ST	1 (0.5)	0 (0.0)	1.000
Mixed infections	23 (11.0)	19 (12.6)	
Total	72 (34.3)	82 (54.7)	< **0.001**

EAEC = enteroaggregative; EHEC = enterohemorrhagic; EIEC = enteroinvasive; EPEC = enteropathogenic; ETEC = enterotoxigenic; LT = heat labile; ST = heat stable.

* *P* value from χ^2^ test or Fisher's exact test, as appropriate; boldface indicates statistical significance.

**Table 4:** The clinical manifestations of viral- and bacterial-associated diarrhea

Characteristic	Viral infection *n* (% or IQR)	Bacterial infection *n* (% or IQR)	Mixed viral/bacterial infection *n* (% or IQR)	*P* value∥
	*N* = 863	*N* = 143	*N* = 60	
Bloody diarrhea	4 (0.5)	12 (8.4)	0 (0)	< **0.001**
Mucoid diarrhea	93 (10.8)	60 (42.0)	16 (26.7)	< **0.001**
Watery diarrhea	766 (86.1)	71 (49.7)	44 (73.3)	< **0.001**
Mild fever	480 (55.6)	73 (51.0)	33 (55.0)	0.319
Severe fever	165 (19.1)	45 (31.5)	4 (6.7)	**0.001**
Dehydration	92 (10.7)	4 (2.8)	6 (10.0)	**0.002**
Vomiting	730 (84.6)	89 (62.2)	46 (76.7)	< **0.001**
Cough	274 (31.7)	38 (26.6)	20 (33.3)	0.242
Abdominal pain	40 (4.6)	30 (21.0)	3 (5.0)	< **0.001**
Anorexia	533 (61.8)	74 (51.7)	38 (63.3)	**0.027**
WBC + stool	191 (22.1)	100 (69.9)	27 (45.0)	< **0.001**
RBC + stool	94 (10.9)	78 (54.5)	16 (26.7)	< **0.001**
Average daily episodes*	4 (3–7)	5 (3–8)	5.5 (3–8.5)	0.414
Maximum daily episodes†	10 (6–13)	10 (6–13)	10 (6–12)	0.904
Length of illness‡	2 (2–3)	2 (1–3)	2 (1–3)	< **0.001**
Length of stay§	5 (3–7)	4 (3–7)	5 (2.5–7)	0.5247

RBC = red blood cells; WBC = white blood cells.

* Average number of diarrheal episodes in a 24-hour period, as reported by the parent/guardian.

† Maximum number of diarrheal episodes in a 24-hour period, as reported by the parent/guardian.

‡ Prior to hospitalization (days).

§ Hospitalization duration (days).

∥ Comparison of viral and bacterial infections only. Fisher's exact, χ^2^ or Mann–Whitney *U* test as appropriate; boldface indicates statistical significance.

**Table 5:** Antimicrobial resistance among *Campylobacter*, *Salmonella*, and *Shigella* from diarrheal cases

Antimicrobial	*Campylobacter*				*Salmonella*						*Shigella*		
	Total (%)	*coli* (%)	*jejuni* (%)	Spp. (%)	Total (%)	Gp B (%)	Gp C (%)	Gp D (%)	Spp. (%)	*ariz* (%)	Total (%)	*flexneri* (%)	*sonnei* (%)
AMP	17/66 (26.3)	5/20 (28)	12/44 (26.5)	0/2 (0)	48/78 (48.1)	36/46 (65.5)	3/10 (27.2)	4/5 (80)	4/15 (18.7)	1/2 (50)	48/62 (77.4)	3/4 (75)	45/58 (77.5)
AMC	2/66 (3)	0/20 (0)	2/44 (4.5)	0/2 (0)	1/76 (1.3)	1/44 (2.3)	0/10 (0)	0/5 (0)	0/15 (0)	0/2 (0)	1/60 (1.7)	0/4 (0)	1/56 (1.8)
CAZ	10/66 (15.1)	5/20 (25)	5/44 (11.3)	0/2 (0)	6/78 (7.7)	5/46 (10.8)	1/10 (10)	0/5 (0)	0/15 (0)	0/2 (0)	1/62 (1.6)	0/4 (0)	1/58 (1.7)
CIP	52/65 (80.0)	20/20 (100)	30/43 (69.7)	0/2 (0)	4/76 (5.3)	4/44 (9.1)	0/10 (0)	0/5 (0)	0/15 (0)	0/2 (0)	1/61 (1.6)	0/4 (0)	1/57 (1.8)
GAT	8/66 (12.1)	2/20 (10.0)	6/44 (13.6)	0/2 (0)	0/78 (0)	0/46 (0)	0/10 (0)	0/5 (0)	0/15 (0)	0/2 (0)	0/62 (0)	0/4 (0)	0/58 (0)
OFL	54/66 (81.8)	20/20 (100)	32/44 (72.7)	2/2 (100)	3/78 (3.8)	2/46 (4.3)	0/10 (0)	1/5 (20)	1/15 (6.7)	0/2 (0)	1/62 (1.6)	0/4 (0)	1/58 (1.7)
CHL	1/66 (1.5)	0/20 (0)	1/44 (2.3)	0/2 (0)	30/77 (38.9)	24/45 (53.3)	2/10 (20)	2/5 (40)	2/15 (13.3)	0/2 (0)	4/61 (6.6)	3/4 (75)	1/57 (1.8)
TMP	51/65 (78.4)	17/20 (85)	32/43 (74.4)	2/2 (100)	30/78 (38.4)	24/46 (52.1)	2/10 (20)	2/5 (40)	2/15 (13.3)	0/2 (0)	55/59 (93.2)	3/4 (75)	52/55 (94.5)
NA	56/66 (84.8)	20/20 (100)	34/44 (77.2)	2/2 (100)	14/78 (17.9)	11/46 (23.9)	0/10 (0)	2/5 (40)	1/15 (6.7)	0/2 (0)	56/62 (90.3)	2/4 (50)	54/58 (93.1)
ERY	5/64 (7.8)	5/20 (25.0)	0/42 (0)	0/2 (0)	−	−	−	−	−	−	−	−	−
CRO	−	−	−	−	10/78 (12.8)	8/46 (17.3)	1/10 (10)	0/5 (0)	1/15 (6.7)	0/2 (0)	45/62 (72.5)	0/4 (0)	45/58 (77.5)

AMC = augmentin; AMP = ampicillin; CAZ = ceftazidime; CHL = chloramphenicol; CIP = ciprofloxacin; CRO = ceftriaxone; ERY = erythromycin; GAT = gatifloxacin; NA = nalidixic acid; OFL = ofloxacin; TMP = trimethoprim.
